# The effect of acupuncture on the day of embryo transfer on the in vitro fertilization outcomes: An RCT

**DOI:** 10.18502/ijrm.v18i3.6719

**Published:** 2020-03-29

**Authors:** Alamtaj Samsami Dehghani, Kaynoosh Homayouni, Zahra Kanannejad, Zeinab Kanannejad

**Affiliations:** ^1^Department of Obstetrics and Gynecology, School of Medicine, Shiraz University of Medical Sciences, Shiraz, Iran.; ^2^Department of Obstetrics and Gynecology, Infertility Research Center, Shiraz University of Medical Sciences, Shiraz, Iran.; ^3^Research Center for Traditional Medicine and History of Medicine, Shiraz University of Medical Sciences, Shiraz, Iran.; ^4^Department of Immunology, Shiraz University of Medical Sciences, Shiraz, Iran.

**Keywords:** Acupuncture, Embryo transfer, In vitro fertilization, Pregnancy rate.

## Abstract

**Background:**

Acupuncture is an adjunct therapy to support infertile women received in vitro fertilization (IVF) treatment; however, the efficacy of this approach needs more evaluation.

**Objective:**

This randomized clinical trial (RCT) study aimed to evaluate the influence of acupuncture on reproductive outcomes in women undergoing IVF treatment.

**Materials and Methods:**

The study was carried out on 186 participants who had undergone IVF treatment in the Mother and Child Hospital between September 2015 and February 2016. Subjects were randomly divided into three groups: Acupuncture 25 min before embryo transfer (ET) (ACU1 group, n = 62), acupuncture 25 min before and after ET (ACU2 group, n = 62), and ET without acupuncture (control group, n = 62). Pregnancy rates (biochemical, clinical, and ongoing) were evaluated and compared between groups.

**Results:**

There were significant differences between the ACU1 group and the control group regarding biochemical (p = 0.005), clinical (p = 0.006), and ongoing (p = 0.007) pregnancies. Also, our results showed that two-session acupuncture (ACU2) lead to a significant reduction in frequency of biochemical (p = 0.002), clinical (p = 0.003), and ongoing (p = 0.01) pregnancy rates when compared to the one-session acupuncture (ACU1). No significant difference was found between the ACU2 and control groups regarding the aforementioned terms (p = 0.50).

**Conclusion:**

Acupuncture 25 min before ET significantly increased the IVF outcomes in women undergoing IVF compared with no acupuncture. Repeating acupuncture 25 min after ET did not improve the IVF outcome.

## 1. Introduction

In vitro fertilization (IVF) is a commonly used technique for the treatment of different types of infertility. Despite many recent technological advances, the average success rates with IVF is still about 20-25% (1). Various methods such as controlled ovarian stimulation, prescription of antioxidants such as Vitamin C or folic acid (2), and acupuncture have been introduced to increase the efficacy of IVF (3). Acupuncture has been applied as a useful therapeutic mean in the treatment of many diseases (4). It has become more popular among western world on account of low side-effects, convenience, and great effect on general well-being. Acupuncture is based on meridians where qi and blood flow within the body. There are specific points alongside these meridians known as acupoints. Acupuncture can regulate the flow of qi and blood in the meridian system by using acupoints stimulation. It regulates the disturbance of the organs' function in the body and restores their normal functions (5). Zhongji (CV3), Guanyuan (CV4), Sanyinjiao (SP6), and Zigong (EX-CA1) are commonly chosen acupoints to treat infertility (6).

The suggested mechanisms that acupuncture affects the women's infertility include: A common sympathoinhibitory effect by increasing the blood flow to the uterus and ovaries, which improves implantation conditions (7) and central stimulation of β-endorphin secretion (8), resulting in gonadotrophin and steroid secretion (9).

Many studies claim positive effect of the acupuncture in the treatment of female infertility. In a prospective, randomized study by Stener-Victorin and co-worker (10), it was shown that electro-acupuncture during oocyte aspiration in IVF treatment can result in a significant higher implantation rate and successful delivery as compared with alfentanil administration. In another prospective randomized trial, Westergaard and colleagues (11) revealed using acupuncture can significantly improve IVF outcome on the day of embryo transfer (ET). In a meta-analysis study of 10 randomized clinical trial (RCT) studies, (6) a significant increase in pregnancy rate (PR) of IVF treatment was considered especially when acupuncture was administered on the day of ET. Paulus and colleagues showed a significantly increased clinical PR in the patients received acupuncture on the day of ET as compared with patients not received acupuncture treatment (7).

However, despite the mentioned studies, the clinical effectiveness of acupuncture is still a matter of debate with some studies like the meta-analysis of El-Toukhy and co-worker (8), disapproving a significant increase in PR when acupuncture was performed around the time of ET.

In the present study, women undergoing IVF treatment were randomly allocated to one of the three groups: no acupuncture (control group), acupuncture 25 min before ET (ACU1), and acupuncture 25 min before and after ET (ACU2 group).

In this study we aimed to investigate the effects of acupuncture on the reproductive outcomes in patients selected for IVF treatment through comparing biochemical pregnancy, clinical pregnancy, and ongoing pregnancy in three studied groups.

## 2. Materials and Methods

### Study design 

This prospective randomized study was carried out in the Mother and Child Hospital, Shiraz University of Medical Sciences, Shiraz, Iran, between September 2015 and February 2016. It was approved by the ethics committee of Shiraz University of Medical Sciences (IR.SUMS.REC.1394.20). A total of 186 infertile women undergoing IVF treatment were recruited in the study. All patients were informed about the aims and practical details of the study, and willingness to participate was confirmed in writing.

The inclusion criteria of the study were the couples' consent and women diagnosed with infertility and undergoing assisted reproductive technology (ART) attempts. Patients who did not achieve ET or did not want to participate further were excluded from the study.

Power calculations (Medcalc software, Mariakerke, Belgium) predicted that 50 patients in each group would require for a significant difference in PR of 10% between no acupuncture and acupuncture based on an expected average PR of 30% per ET. Considering 20% decrease in the patients, approximately 186 patients were required to provide the study with a power of 80% and a P-value of 0.05.

### Randomization

One hundred and eighty-six patients were randomly divided into three groups: (1) no acupuncture (control group, n = 62); (2) acupuncture 25 min before ET (ACU1 group, n = 62); and (3) acupuncture 25 min before and after ET (ACU2 group, n = 62).

### Acupuncture

In this study, we used small stainless steel needles (25*13 mm, Hanyi needle) for acupuncture at the following points: Ht.7 (Shenmen), PC.6 (Neiguan), Ren.6 (qihai), Du.20 (Baihui), SP.6 (Sanyinjiao), and Ren.4 (Guanyuan). Treatments were performed by an acupuncturist.

In the ACU1 group, needles were inserted in the mentioned acupuncture point 25 min before ET in one session. For the ACU2 group, the acupuncture protocol was the same as the ACU1 group, but this group received acupuncture in two sessions, 25 min before and after the ET. Needles were inserted into the mentioned acupoints until Deqi needle-sensation was obtained. The degree of needle-sensation is called Deqi and is a feeling of soreness, numbness, distension, or pain. The control group just received the clinic's routine procedure for IVF treatment.

### IVF protocol

At first, all patients received long down-regulation protocol, with a GnRH agonist (nafarelin 0.4 mg daily). This protocol begun of the day 21 of the previous cycle until the day of hCG injection. Ovarian stimulation was induced with recombinant FSH or hMG. Ovulation triggering was induced by hCG (10,000 IU) injection when at least three follicles had a diameter of 18 mm as well as an adequate serum estradiol concentration. Transvaginal oocyte retrieval was done under ultrasound guidance, 35 hr after hCG administration. Three embryos were transferred into the uterus 2 to 3 days after the oocyte retrieval. Also, all patients were treated with progesterone (200 mg three times daily) after the oocyte retrieval step. Patients with positive biochemical pregnancies (serum hCG > 10 IU/L) (12) were scanned by transvaginal ultrasound. Positive clinical pregnancy was confirmed 6 week after ET by ultrasound examination and the presence of a fetal sac (7), also the ongoing PR was defined as detection of confirmed pregnancies by ultrasound scan and continued for at least 21 wk after ET (13).

### Ethical consideration 

This trial was approved by the ethics committee of the Shiraz University of Medical Sciences (no: IR.SUMS.REC.1394.20) and by the Iranian Registry of Clinical Trials (IRCT20150511022206N4). All participants provided oral and written informed consent.

### Statistical analysis

Fisher's exact test was performed to compare qualitative variables and one-way ANOVA to compare quantitative variables. Data were analyzed using statistical package SPSS software (Statistical Package for the Social Sciences, version 16.0, SPSS, Inc., Chicago, IL). P < 0.05 were considered significant.

## 3. Results 

Descriptive data on both acupuncture and control groups were shown in Table I. There were no statistically significant differences between the three studied groups regarding patients' age, BMI, smoking, the number of transferred embryo, duration of infertility, primary or secondary infertility, previous IVF attempt, endometrial thickness, and causes of infertility.

The reproductive outcomes in the three groups are shown in Table II. The analysis showed that biochemical PR for the ACU1 group was significantly higher than the control (p = 0.005) and ACU2 (p = 0.002) groups. Furthermore, the rates of clinical pregnancies and ongoing pregnancy were significantly higher in the ACU1 group than in the control (p = 0.006, p = 0.007, respectively) and ACU2 groups (p = 0.003, p = 0.01, respectively).

**Table 1:** Descriptive data of acupuncture and control groups (Mean ± SD)


**Characteristic**		**Control (n = 62)**	**ACU1 (n = 62)**	**ACU2 (n = 62)**	**P-value**
**Age of patients (years)**		31.5 ± 5.4	32.1 ± 5.9	32.9 ± 4.8	0.45
**BMI (kg/m${}^{2}$)**		26.3 ± 3.9	25.1 ± 3.3	25.2 ± 3.8	0.30
**Smoking (%)**		3.2	3.2	3.2	0.60
**No. of transferred embryo**		2.5 ± 0.6	2.5 ± 0.6	2.5 ± 0.5	0.99
**Duration of infertility (years)**		5.4 ± 4	5.7 ± 3.4	5.1 ± 3.1	0.94
**Primary infertility (%)**		84.2	78.9	81.2	0.38
**Secondary infertility (%)**		10.5	21	18.7	0.32
**Previous IVF attempt**		1.6 ± 1.6	1.4 ± 1.2	1.2 ± 0.4	0.35
**Endometrial thickness**		7.6 ± 0.7	8.1 ± 0.5	8.04 ± 0.6	0.06
	**Male**	57.8	47.3	50	
	**Female**	15.7	26.3	18.7	0.57
**Causes of infertility (%)**	**Unexplained**	26.3	26.3	31.2	
	BMI: Body mass index; IVF: In vitro fertilization; NS: Not significant ANOVA and Fisher exact test were used for data analysis P < 0.05 was considered to be statistically significant					

**Table 2:** Reproductive outcomes per ET


**Reproductive outcome**	**Control (n = 62)**	**ACU1 (n = 62)**	**ACU2 (n = 62)**	**Total (n = 186)**	**P${}^{a}$**	**P${}^{b}$**	**P${}^{c}$**
**Biochemical pregnancy**	14/62 (22.6%)	30/62 (48.4%)	13/62 (21%)	57/186 (30.6%)	0.005	0.002	0.50
**Clinical pregnancy**	12/62 (19.3%)	27/62 (43.5%)	11/62 (17.7%)	50/186 (26.8%)	0.006	0.003	0.50
**Ongoing pregnancy**	9/62 (14.5%)	22/62 (35.4%)	7/62 (11.3%)	38/186 (20.5%)	0.007	0.01	0.50
ACU: Acupuncture; NS: Not significant Fisher's exact test (two-tailed) was used for data analysis P < 0.05 was considered to be statistically significant ${}^{a}$Significant difference between control and ACU1; ${}^{b}$Significant difference between ACU1 and ACU2; ${}^{c}$Significant difference between control and ACU2							

## 4. Discussion 

In this prospective, randomized study, we showed that acupuncture treatment 25 min before ET had a positive effect on the reproductive outcomes in the women undergoing IVF treatment. This part of our results confirmed other prospective, randomized studies that previously reported the beneficial effect of acupuncture in reproductive outcome (3, 7, 11, 14, 15). However, current study showed that the reproductive outcomes in women who received acupuncture twice 25 min before and after ET were not significantly different in comparison to the control group. Similar to our results, Westergaard (11) and Benson (14) showed that acupuncture before ET significantly increased IVF outcomes, but repeating acupuncture after ET did not improve the IVF outcomes. Contrary to our finding, Paulus and colleagues present that repeated acupuncture (25 min before and after ET) improved IVF outcome in women undergoing the treatment (7).

Acupuncture may improve the IVF outcome possibly by decreasing blood flow impedance and increasing the uterine blood flow (16), inhibiting uterine motility (17), reducing depression, anxiety, and stress (18, 19), and modulating the immune factors, especially cytokines (20).

The first randomized, controlled, prospective study on the effect of acupuncture on PR in women undergoing IVF treatment was published by Paulus and colleagues. They showed that acupuncture had a beneficial effect on IVF outcome. A meta-analysis study by Manheimer and co-worker on women undergoing IVF treatment showed the association of acupuncture at the time of ET with significant improvement in clinical PR, ongoing PR, and live birth rate (21).

On the other hand, some studies have shown that acupuncture had no obvious effect on the IVF outcomes (22-24).

In short, the reasons for the difference between the results of various studies may be as follows: First, different studies have used different acupuncture points; as an example, the acupuncture points used in the study by Andersen and colleagues included DU20, M29, KS6, Mp8 and Le3 before the ET and DU20, Co4, Mp10, M36, and Mp6 after it (25), whereas, we used the following acupuncture points: Ht.7 (Shenmen), PC.6 (Neiguan), Ren.6 (qihai), Du.20 (Baihui), SP.6 (Sanyinjiao), and Ren.4 (Guanyuan). Second, in different studies, various methodologies were used; for example, Andersen and colleagues compared the results of IVF outcomes in patients undergoing acupuncture 30 min before and after ET with patients receiving placebo acupuncture (25). However, we compared the IVF outcomes in patients undergoing acupuncture 25 min before ET with patients receiving acupuncture 25 min before and after ET.

The chief innovation of the current study was the comparison of the effects of one-session acupuncture (25 min before ET) with two-sessions acupunctures (25 min before and after ET) on IVF outcomes for the first time; however, there were some limitations in the present study. The uterine, ovarian, and the endometrial blood flows during ET or after acupuncture and the related hormonal factors (hormones, peptide growth factors) were not assessed. Also, a study with larger samples may be needed to confirm our results.

## 5. Conclusion

Our findings showed that acupuncture 25 min before ET significantly increased the IVF outcome in women undergoing IVF, but repeating acupuncture 25 min after ET did not improve the IVF outcome in comparison to the control group.

##  Conflict of Interest

The authors declare that there is no conflict of interest.
